# A response surface methodology approach to improve nitrogen use efficiency in maize by an optimal mycorrhiza-to-*Bacillus* co-inoculation rate

**DOI:** 10.3389/fpls.2022.956391

**Published:** 2022-08-11

**Authors:** Paola Ganugi, Andrea Fiorini, Gabriele Rocchetti, Paolo Bonini, Vincenzo Tabaglio, Luigi Lucini

**Affiliations:** ^1^Department for Sustainable Food Process, Università Cattolica del Sacro Cuore, Piacenza, Italy; ^2^Department of Sustainable Crop Production, Università Cattolica del Sacro Cuore, Piacenza, Italy; ^3^Department of Animal Science, Food and Nutrition, Università Cattolica del Sacro Cuore, Piacenza, Italy; ^4^oloBion-OMICS LIFE LAB, Barcelona, Spain

**Keywords:** biostimulants, *Bacillus megaterium*, *Rhizoglomus irregulare*, *Funneliformis mosseae*, nutrients uptake, plant growth promoting rhizobacteria

## Abstract

Co-inoculation of arbuscular mycorrhizal fungi (AMF) and bacteria can synergically and potentially increase nitrogen use efficiency (NUE) in plants, thus, reducing nitrogen (N) fertilizers use and their environmental impact. However, limited research is available on AMF-bacteria interaction, and the definition of synergisms or antagonistic effects is unexplored. In this study, we adopted a response surface methodology (RSM) to assess the optimal combination of AMF (*Rhizoglomus irregulare and Funneliformis mosseae*) and *Bacillus megaterium* (a PGPR—plant growth promoting rhizobacteria) formulations to maximize agronomical and chemical parameters linked to N utilization in maize (*Zea mays* L.). The fitted mathematical models, and also 3D response surface and contour plots, allowed us to determine the optimal AMF and bacterial doses, which are approximately accorded to 2.1 kg ha^–1^ of both formulations. These levels provided the maximum values of SPAD, aspartate, and glutamate. On the contrary, agronomic parameters were not affected, except for the nitrogen harvest index (NHI), which was slightly affected (*p*-value of < 0.10) and indicated a higher N accumulation in grain following inoculation with 4.1 and 0.1 kg ha^–1^ of AMF and *B. megaterium*, respectively. Nonetheless, the identification of the saddle points for asparagine and the tendency to differently allocate N when AMF or PGPR were used alone, pointed out the complexity of microorganism interaction and suggests the need for further investigations aimed at unraveling the mechanisms underlying this symbiosis.

## Introduction

Nitrogen (N) represents a major nutrient for plants, being an essential component of proteins, nucleotides, chlorophyll, and a broad range of secondary metabolites. Cereals grains, providing 60% of the food necessary to feed the world’s population, require significant inputs of N to achieve optimum yields. Nevertheless, N availability can represent a limiting condition since nitrate (NO_3_^–^) and ammonium (NH_4_^+^), representing the readily available N pool, account for only 2% of total soil N content (Moreau et al., 2019).

At the same time, the increasing use of synthetic N fertilizers over the last decades has posed concerns about the contamination of surface and groundwater bodies by nitrate, the impairment of biodiversity, and the emission of greenhouse gases (Ahmed et al., 2017). In line with the EU “Farm to fork” strategy, which aims at increasing the agricultural land managed under organic farming by 25%, scientific efforts are being done to maintain (or even increase) crop productivity by efficiently using organic fertilizers, which have been recognized to increase N losses very often, especially if applied inappropriately (Maris et al., 2021). Such an increase in N use efficiency can significantly minimize N losses and the consequent adverse impacts on ecosystems while decreasing costs for organic fertilizers (Galloway et al., 2014).

All the pathways of N cycling in soils mainly depend on the edaphic conditions, agronomic management, climate, crop genetics, and finally determines N availability, transfer, transformation, and losses (Congreves et al., 2021). Many scientific contributions have reported best agronomic practices and breeding solutions to enhance NUE, commonly defined as the plant biomass accumulation per unit of soil N available (Peng et al., 2006; van Bueren and Struik, 2017; Anas et al., 2020). N assimilation by plants involves the GS–GOGAT pathway, for which glutamine, asparagine, glutamate, and aspartate are upstream key intermediates; once incorporated into organic compounds, N is then distributed to a broad range of different N-containing compounds. Over the last two decades, biotechnological engineering of the amino acid metabolism has led to promising results for the improvement of NUE, especially the content of glutamine, asparagine, glutamate, and aspartate has been adopted as an interesting target to carry out these studies, being involved in plant N utilization and storage after assimilation (Dellero, 2020; The et al., 2021). Recently, especially in a framework of sustainable crop production, the inoculation with beneficial microorganisms has gained importance in such plant NUE increase (Di Benedetto et al., 2017; Verzeaux et al., 2017; Dalla Costa et al., 2021).

Arbuscular mycorrhizal fungi (AMF) have been reported to increase NUE by developing the symbiotic association with most terrestrial plants, favoring access to N uptake in a larger soil volume (Verzeaux et al., 2017). Interestingly, the specific up-regulation of NO_3_^–^ and NH_4_^+^ transporters has been observed in AMF colonized roots compared with the non-colonized plants (Courty et al., 2015; Garcia et al., 2016). In cereals such as sorghum, maize, and rice, AMT3.1 plant NH_4_^+^ transporter transcripts were specifically up-regulated following the mycorrhizal colonization (Koegel et al., 2017). Similarly, higher NUE has been observed following beneficial associations with N2-fixing bacteria such as Rhizobia, *Frankia* sp., and Cyanobacteria, and some other diazotrophs like *Azospirillum* spp., *Herbaspirillum* spp., and *Paenibacillus* spp. Interestingly, a community of mycorrhizosphere bacteria living strictly associated with AMF has been suggested to encompass improved crop performances, acting synergistically with AMF (Oldroyd et al., 2011; Santi et al., 2013; Udvardi and Poole, 2013; Agnolucci et al., 2015; Kollah et al., 2016; Mus et al., 2016; Rosenblueth et al., 2018). In this regard, a high degree of specificity between bacteria and AMF has been proposed, and this tripartite association can potentially increase NUE in plants (Tajini et al., 2012; Giovannini et al., 2020; Paul et al., 2020).

The dynamic assembly of the rhizosphere microbial community depends on a large set of factors and is driven by an intricate set of belowground chemical communications (van Dam and Bouwmeester, 2016; Qu et al., 2020). However, very little information is available in the literature regarding optimizing AMF-bacteria co-inoculation to manipulate rhizomicrobiome and improve plant performance.

The present study aimed at optimizing the co-inoculation between mycorrhiza and *Bacillus megaterium* using the response surface methodology (RSM), with reference to enhanced N utilization in maize (*Zea mays* L.). The RSM approach has been chosen to account for the interaction(s) between the fungal and bacterial inoculum in the framework of the complex rhizosphere community. Similarly, different plant-based indices of NUE (i.e., NHI and NUtE; López-Bellido and López-Bellido, 2001) and yield of maize have been considered to account for the different assimilation, metabolization, mobilization processes, as well as the translocation to reproductive portions, to overcome the temporal and spatial edges of NUE indices (Congreves et al., 2021).

## Materials and methods

### Experimental site and microorganism inoculation

The field experiment was conducted over one maize cropping season—between May 2021 and September 2021—at the CERZOO experimental research station in Piacenza (45°00′21.6′′N, 9°42′27.1′′E; altitude 68 m a.s.l.), Northern Italy. At the experimental site, the climate is temperate (Cfa following Köppen classification), with an average annual temperature of 13.2°C and cumulative annual precipitation of 837 mm (20-yr data). The soil is a fine, mixed, mesic Udertic Haplustalf based on the Keys to Soil Taxonomy (Soil Survey Staff 2014). The physicochemical properties of soil (0–30 cm soil layer) measured before starting the experiment were: organic matter content 27 g kg^–1^; pH H_2_O 7.8; bulk density 1.36 g cm^–3^; sand 127 g kg^–1^; silt 445 g kg^–1^; clay 428 g kg^–1^; soil total N 1.3 g kg^–1^; available P (Olsen) 32 mg kg^–1^; exchangeable K (NH_4_^+^ Ac) 294 mg kg^–1^, and cation exchange capacity 30 cmol^+^ kg^–1^. Biostimulant treatments consisted of a seed dressing with the powder AMF-based product Aegis Sym irriga^®^ (*Rhizoglomus irregulare* BEG72 and *Funneliformis mosseae* BEG234, 700 sp g^–1^ each species) formulation, or the PGPR Bactrium^®^ (*Bacillus megaterium* BM77 e BM06, 5 × 10^9^ CFU/g each species) liquid formulation, all from Athens, Agrotecnologia Naturales SL (Tarragona, Spain). The seed dressing was homogenized with maize seeds using an automated mixer.

To assess the effect of different doses of AMF and bacteria on yield and NUE in maize, nine different types of seed dressing were prepared as all the possible combinations of the three doses of AMF (low: 0.1 kg ha^–1^; medium: 2.1 kg ha^–1^; and high: 4.1 kg ha^–1^) and three doses of *B. megaterium* (low: 0.1 kg ha^–1^; medium: 2.1 kg ha^–1^; and high: 4.1 kg ha^–1^). As a result, the present field experiment was set up as a randomized complete block (RCB) design with 9 treatments and 4 replicates (blocks). Each plot was 28 m^2^: 10 m long and 2.8 m wide (4 maize rows at a 0.7 m inter-row distance). Before planting maize, the seed dressing was carried out in the Lab separately for each treatment. In brief, each level of the 3 × 3 AMF-*B. megaterium* doses were manually mixed with maize seeds (at around 82,000 seeds ha^–1^). Then, maize was planted with a common two-row plot seeder on 7 May 2021. Maize cropping management followed principles reported by the regulation (EU) 2018/848 on the organic farming production methods. To represent local practice under organic farming, a 170 kg N ha^–1^ slurry distribution was applied to all the plots before soil tillage (autumn 2020), and no fertilizer was applied during the cropping cycle of maize. To prevent water stress, maize was sprinkler-irrigated three times at doses of 30, 40, and 45 mm. Harvesting took place on 20 September 2021 with a plot-scale combination.

### SPAD measurement

On 13 August, relative chlorophyll concentrations, defined as SPAD value, were estimated between 02:00 p.m. and 03:00 p.m. using a SPAD-502 chlorophyll meter (Minolta, Tokyo, Japan). The average value of five measurements of three leaves was taken as the maize SPAD value per plot.

### Asparagine, glutamine, aspartate, and glutamate content in leaves

On 13 August, the ninth leaf of five plants per plot was sampled and stored at −20°C. The frozen samples were used to determine the content of four amino acids, namely, asparagine, glutamine, aspartate, and glutamate.

Initially, the five leaves per plot were ground together with liquid nitrogen using a pestle and mortar to obtain a homogeneous and representative final sample. Two aliquots (2 × 1 g) per plot were extracted in 10 ml of 50% methanol, 50% deionized water, and 0.01% formic acid using an Ultra-Turrax (Ika T-25, Staufen, Germany) and successively centrifuged (12,000 × g).

The different extracts were diluted 1,000-fold with deionized water and then filtered in the HPAEC vials using 0.20 μm syringe filters until instrumental analysis. The amino acid content of the hydroalcoholic leaf extracts was then investigated through High-Performance Anion Exchange Chromatography with Pulsed Amperometric Detection (HPAEC–PAD). The analyses were carried out on a Dionex ICS-5000 + instrument (Thermo Fisher Scientific, Waltham, the United States) provided with an electrochemical cell consisting of a gold-working electrode and a pH–Ag/AgCl reference electrode. The separation was performed using a Thermo Scientific™ Dionex™ AMINOPAC™ PA10 Analytical column (2 × 250 mm). The chromatographic runs were executed in a total time of 80 min, with multi-gradient elution consisting of a mobile phase of deionized water (eluent A), 250 mM aqueous sodium hydroxide (eluent B), and 1 M sodium acetate solution (eluent C). The flow rate was 0.25 ml/min, and the injection volume was 25 μl, with a column and detector compartment temperature at 30°C. Gradient elution consisted of 0–12 min with 80% of eluent A and 20% of eluent B; 12–16 min with 68% of eluent A and 32% of eluent B; 16–40 min of 36% of eluent A, 24% of eluent B, and 40% of eluent C. Also, a 40 min of equilibration phase was considered, sing 80% of eluent A and 20% of eluent B. The amino acids were detected by an ICS-5000 + electrochemical detector in integrated pulsed amperometric detection mode applying the following waveform potentials and durations: E1 = 0.13 V (t1 = 0.40 s), E2 = 0.33 V (t2 = 0.210 s), E3 = 0.55 V (t3 = 0.460 s), E4 = 0.33 V (t4 = 0.560 s), E5 = −1.67 V (t5 = 0.580 s), E6 = 0.93 V (t6 = 0.590 s), and E7 = 0.13 V (t7 = 0.600 s). For the quantification step, a calibration curve (R2 > 0.98) of the four amino acids was prepared by appropriately diluting an amino acid standard mix (2.5 μmol/ml in 0.1M HCl solution, provided by Merck, Darmstadt, Germany), considering the following concentration range: 25-10-5-2.5-1 μM (Supplementary Figure 2). The chromatographic system was controlled through the Thermo Scientific™ Dionex™ Chromeleon™ software version 7.0 for the instrumentation command, chromatograms acquisition, and processing.

### Maize yield and agronomic nitrogen use efficiency indices

At the BBCH 89, maize yield was measured by manually harvesting 8 m^2^ per single plot. Plants were weighed and separated into grain and biomass (stalks). A 100-g sub-sample of each grain and biomass sample was oven-dried at 65°C until constant weight to measure dry matter content. Grain and biomass N-uptake were calculated by multiplying grain and biomass yield by their N-concentrations, determined by the Dumas combustion method with an elemental analyzer varioMax C:N (VarioMax C:NS, Elementar, Germany).

The two following N-efficiency parameters were calculated for each treatment according to López-Bellido and López-Bellido (2001): (i) N harvest index (NHI;%) as the ratio of N in grain to N in total plant biomass; and (ii) N-utilization efficiency (NUtE; kg kg^–1^) as the ratio of grain yield to total plant N-uptake.

### Statistical analysis

Data analysis was performed with Rstudio 3.6.1 software (R Core Team, 2013). On each NUE parameter, two different two-way ANOVA (*p* < 0.05) were carried out. The first ANOVA was focused on the differences between treatments, thus, the combination of AMF and bacterial doses, as well as the block, were considered as factors, respectively, represented by 9 and 3 levels. The response profiles not impacted by the microbial inoculations were plotted as box plots using the *ggplot* package to represent the data distribution according to treatments (Wickham and Chang, 2007). Successively, the second ANOVA was performed to study the effects and the interactions of the AMF and bacteria factors.

After that, response-surface analysis was performed with the *rsm* package (Lenth, 2009). According to Kumar et al. (2020), the face-centered composite design (FCDD) was used and two independent variables with three levels were selected: AMF (x1: 0.1 kg ha^–1^; 2.1 kg ha^–1^; 4.1 kg ha^–1^) and B. megaterium (x2: 0.1 kg ha^–1^; 2.1 kg ha^–1^; 4.1 kg ha^–1^). A full factorial design of 27 experimental runs with 12 cube points, 12 axial points, and 3 center points was chosen (Table 1), while SPAD values, amino acid concentrations, and other NUE parameters were considered response profiles for modeling. The experimental runs were randomized to minimize the effect of unexpected variability on the observed responses. The following second-order quadratic model was used to develop the optimization and predictive model:


(1) Y=β+0(βxi*)1+(βxj*)2+(Bxi⁢j*x1*)2+(βxi*)12+(βj*x)22;

**TABLE 1:** RSM–CCD matrix for response surface analysis on the maize experiment.

Run	Coded label		Actual label	
		
	Factor x_1_	Factor x_2_	Factor x_1_	Factor x_2_
	AMF dose	*B. megaterium* dose	AMF dose	*B. megaterium* dose
	(ka ha^–1^)	(ka ha^–1^)	(ka ha^–1^)	(ka ha^–1^)
1	−1	−1	0.1	0.1
2	+1	0	4.1	2.1
3	−1	0	0.1	2.1
4	+1	+1	4.1	4.1
5	0	+1	2.1	4.1
6	0	0	2.1	2.1
7	0	−1	2.1	0.1
8	+1	−1	4.1	0.1
9	+1	−1	4.1	0.1
10	+1	0	4.1	2.1
11	+1	+1	4.1	4.1
12	−1	0	0.1	2.1
13	0	0	2.1	2.1
14	−1	+1	0.1	4.1
15	0	+1	2.1	4.1
16	+1	−1	4.1	0.1
17	−1	−1	0.1	0.1
18	+1	0	4.1	2.1
19	−1	+1	0.1	4.1
20	0	−1	2.1	0.1
21	−1	0	0.1	2.1
22	−1	−1	0.1	0.1
23	0	+1	2.1	4.1
24	0	−1	2.1	0.1
25	−1	+1	0.1	4.1
26	0	0	2.1	2.1
27	+1	+1	4.1	4.1

where Y is the predicted response profile, β_0_, β_*i*_, β_*j*_, and β_*ij*_ are the interactive regression coefficients, and x_1_ and x_2_ are the AMF and *B. megaterium* doses, respectively.

## Results

Analysis of variance revealed a significant effect of the treatments on maize in terms of SPAD value and amino acid concentrations (Table 2). Interestingly, the highest value of SPAD (62.95 ± 1.41) was found with the intermediate dose of AMF and *B. megaterium*, corresponding to 2.1 kg ha^–1^ of each inoculum. Similarly, the same result was obtained for aspartate, whose highest concentration (0.81 ± 0.38 μM) was registered at the same treatment level, showing a content 4.7- and 3.5-folds higher than those, respectively, registered with the lowest (0.1 kg ha^–1^) and the highest (4.1 kg ha^–1^) doses of both inocula. Surprisingly, asparagine and glutamine concentrations reached the maximum concentration (respectively, 2.95 ± 0.52 and 0.86 ± 0.13 μM) at 2.1 + 2.1 kg ha^–1^ AMF-*B. Megaterium* combination, then decreased with the increasing dosage of AMF and raised again at the highest levels of mycorrhizal and bacterial inocula. At final, the inoculum with 2.1 + 4.1 kg ha^–1^ of AMF and *B. megaterium* combined reflected a higher glutamate content (0.82 ± 0.14 μM).

**TABLE 2:** Two−way ANOVA on SPAD value, amino acid concentrations, and agronomic parameters observed in the maize crop following AMF and *B. megaterium* treatment.

Treatment													
x1 + x2	SPAD value	Aspartate	Glutamate	Asparagine	Glutamine	Biomass	Biomass N conc.	Biomass N uptake	Grain N conc.	Grain N uptake	Grain yield	NHI	NUtE
(kg ha^–^^1^)		(μ M)	(μ M)	(μ M)	(μ M)	(Mg ha^–^^1^)	(%)	(kg ha^–^^1^)	(%)	(kg ha^–^^1^)	(Mg ha^–^^1^)		
0.1 + 0.1	52.89 ± 0.61	0.17 ± 0.14	0.25 ± 0.07	1.57 ± 0.20	0.20 ± 0.11	8.09 ± 0.89	0.72 ± 0.07	58.8 ± 13.64	1.39 ± 0.11	132.23 ± 21.88	9.46 ± 0.85	69.36 ± 1.76	50.14 ± 4.73
0.1 + 2.1	57.01 ± 0.58	0.25 ± 0.11	0.37 ± 0.18	1.90 ± 0.47	0.35 ± 0.07	10.62 ± 0.44	0.79 ± 0.03	84.16 ± 7.21	1.49 ± 0.14	177.59 ± 23.46	11.85 ± 0.64	67.76 ± 1.08	45.56 ± 3.57
0.1 + 4.1	55.38 ± 1.01	0.32 ± 0.04	0.42 ± 0.05	2.08 ± 0.37	0.61 ± 0.04	9.16 ± 1.29	0.76 ± 0.10	69.52 ± 14.89	1.51 ± 0.16	179.71 ± 36.71	11.83 ± 1.51	72.13 ± 1.84	48.04 ± 4.49
2.1 + 0.1	58.01 ± 0.59	0.44 ± 0.35	0.54 ± 0.11	3.27 ± 0.16	0.66 ± 0.15	9.25 ± 1.14	0.8 ± 0.11	73.54 ± 8.87	1.5 ± 0.09	158.79 ± 14.48	10.58 ± 0.93	68.33 ± 3.29	45.49 ± 0.82
2.1 + 2.1	62.95 ± 1.41	0.81 ± 0.38	0.60 ± 0.22	2.95 ± 0.52	0.86 ± 0.13	11.73 ± 2.92	0.75 ± 0.10	87.02 ± 23.38	1.43 ± 0.10	170.09 ± 5.76	11.94 ± 1.27	66.55 ± 5.46	46.45 ± 1.14
2.1 + 4.1	59.93 ± 1.31	0.64 ± 0.17	0.82 ± 0.14	2.79 ± 0.33	1.18 ± 0.17	12.13 ± 3.12	0.71 ± 0.07	87.16 ± 34.15	1.44 ± 0.06	168.86 ± 26.49	11.68 ± 1.80	66.74 ± 5.24	46.22 ± 3.92
4.1 + 0.1	55.12 ± 0.55	0.46 ± 0.18	0.41 ± 0.24	2.66 ± 0.08	1.14 ± 0.15	9.39 ± 0.76	0.69 ± 0.09	64.36 ± 6.56	1.46 ± 0.15	178.73 ± 27.49	12.25 ± 1.09	73.43 ± 1.23	50.73 ± 4.99
4.1 + 2.1	58.98 ± 1.14	0.34 ± 0.16	0.68 ± 0.14	2.07 ± 0.79	0.66 ± 0.05	9.21 ± 1.63	0.72 ± 0.12	67.53 ± 23.61	1.34 ± 0.14	151.67 ± 32.63	11.25 ± 1.63	69.62 ± 4.64	52.49 ± 9.27
4.1 + 4.1	56.03 ± 0.95	0.23 ± 0.01	0.47 ± 0.16	2.52 ± 0.50	0.78 ± 0.14	11.79 ± 0.68	0.8 ± 0.17	95.04 ± 24.55	1.42 ± 0.15	150.32 ± 15.80	10.78 ± 2.23	61.48 ± 8.64	44.23 ± 10.97
**Significance(*p*-value)**													
**Two-wayANOVA**													
**Treatment**	**<2E**−**16**	**0.03**	**0.01**	**2.00E**−**03**	**3.11E**−**07**	0.27	0.86	0.37	0.83	0.33	0.38	0.07	0.71
**Block**	0.98	0.33	0.31	0.18	0.45	0.56	0.57	0.43	0.85	0.54	0.42	0.06	0.58
**Two way ANOVA**													
**AMF dose**	**7.74E**−**03**	**2.52E**−**03**	**2.52E**−**03**	**1.10E**−**04**	**6.37E**−**08**	0.41	0.66	0.60	0.28	0.79	0.55	0.43	0.64
**Bacterial dose**	**3.55E**−**03**	0.53	0.06	0.61	**1.68E**−**03**	**0.045**	0.71	**0.05**	0.86	0.38	0.29	8.2E−02	0.34
AMF dose * Bacterial dose	0.28	0.25	0.29	0.24	**4.40E**−**05**	0.58	0.53	0.38	0.26	9.07E−03	**0.02**	**5.79E**−**03**	0.51

Values are presented as mean ± standard error (SE). Bold values denote statistical significance at the *p* < 0.05 level. The “*” symbolizes the interaction of the two factors (AMF and bacteria) within ANOVA.

**FIGURE 1:**
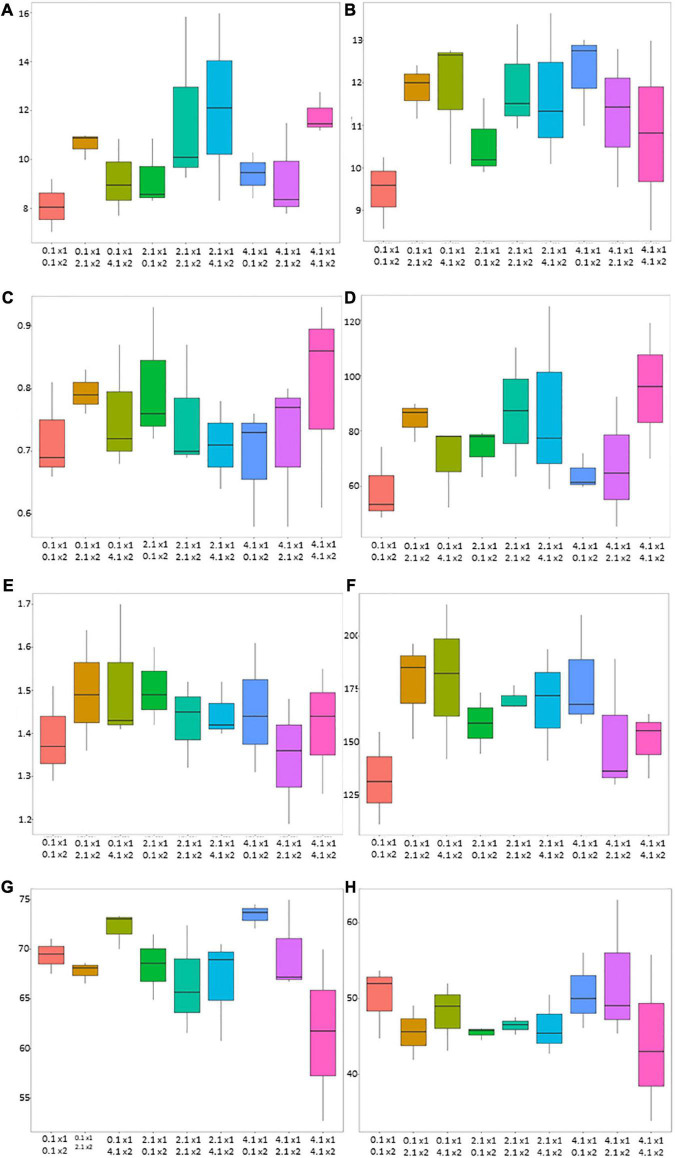
Box plot diagrams of agronomic parameters for AMF and *Bacillus megaterium* treatments on maize (*Zea mays* L.). The *X*-axis describes the combination of AMF and *B. megaterium* doses (0.1 kg ha^–1^, 2.1 kg ha^–1^, and 4.1 kg ha^–1^) while the *Y*-axis represents the values for maize biomass (mg ha^–1^) **(A)**, grain yield (mg ha^–1^) **(B)**, biomass nitrogen concentration (%) **(C)**, biomass nitrogen-uptake (kg ha^–1^) **(D)**, grain nitrogen concentration (%) **(E)**, grain nitrogen-uptake (kg ha^–1^) **(F)**, NHI **(G)**, and NutE **(H)**. Each box line corresponds to the median of the data while the ends of the box show the upper (Q3) and lower quartiles.

However, the ANOVA did not reveal a significant effect of the treatments for the considered agronomic parameters, except for NHI, which seemed to be slightly affected (*p*-value of < 0.10) and indicated a higher N accumulation in grain following 4.1 and 0.1 kg ha^–1^ of AMF and *B. megaterium* inoculation (Table 2). Box plots (Figure 1) provided high-level information regarding data symmetry, variance, and outliers, allowing comparisons between different treatments.

Overall, biomass and biomass N-uptake values showed a similar trend, with 2.1 + 2.1 kg ha^–1^, 2.1 + 4.1 kg ha^–1^, and 4.1 + 4.1 kg ha^–1^ doses of mycorrhizal and bacterial inocula that tended to increase maize performances. Biomass N-concentration tended to be the highest under 0.1 + 2.1 kg ha^–1^, 2.1 + 0.1 kg ha^–1^, and 4.1 + 4.1 kg ha^–1^ doses of mycorrhizal and bacterial inocula.

Grain yield values showed a tendency to be increased under 0.1 + 2.1/4.1 kg ha^–1^, 2.1 + 2.1 kg ha^–1^, and 4.1 + 0.1 kg ha^–1^ doses. Moreover, grain N-concentration tended to be higher (1.51 ± 0.16%) in the absence of mycorrhizal inoculation (0.1 + 4.1 kg ha^–1^ of AMF and *B. megaterium*). At last, the highest NUtE value was obtained with 4.1 kg ha^–1^ and 2.1 kg ha^–1^, respectively, of AMF and *B. megaterium* dose.

A prediction model was developed for those indices being significantly affected (*p*-value of < 0.05) by AMF and *B. megaterium* treatment. The quadratic equations of the final models are given below Eqs. 1–5:


(2) Y=SPAD62.57+0.81x+10.89x-2-0.39xx1-24.39x-123.42x22

(3) Y=ASPARTATE0.69+0.05x+10.02x-20.09xx1-20.33x-120.09x22

(4) Y=GLUTAMATE0.69+0.09x+10.08x-20.03xx1-20.22x-120.07x22

(5) Y=ASPARAGINE2.89+0.28x-10.02x-20.16xx1-20.87x+120.17x22

(6) Y=GLUTAMINE0.80+0.23x+10.09x-20.19xx1-20.27x+120.14x22

Regarding the SPAD value, the model showed a high coefficient of determination (*R*^2^ = 0.9) and a high *F*-value (224.9), while the *p*-value was < 0.05 and no significant lack of fit values (> 0.05) were found. Similarly, a high-predictive model was found for glutamine concentration, exhibiting *R*^2^ = 0.8, *F*-value = 8.75, and a *p*-value of < 0.05. Glutamate, asparagine, and aspartate models showed lower *R*^2^, being 0.5, 0.6, and 0.4, respectively, corresponding to 4.3, 6.4, and 3.9 *F*-values. However, in all the three cases, the *p*-value was < 0.05, and a significant lack of fit was not provided (> 0.05).

**FIGURE 2:**
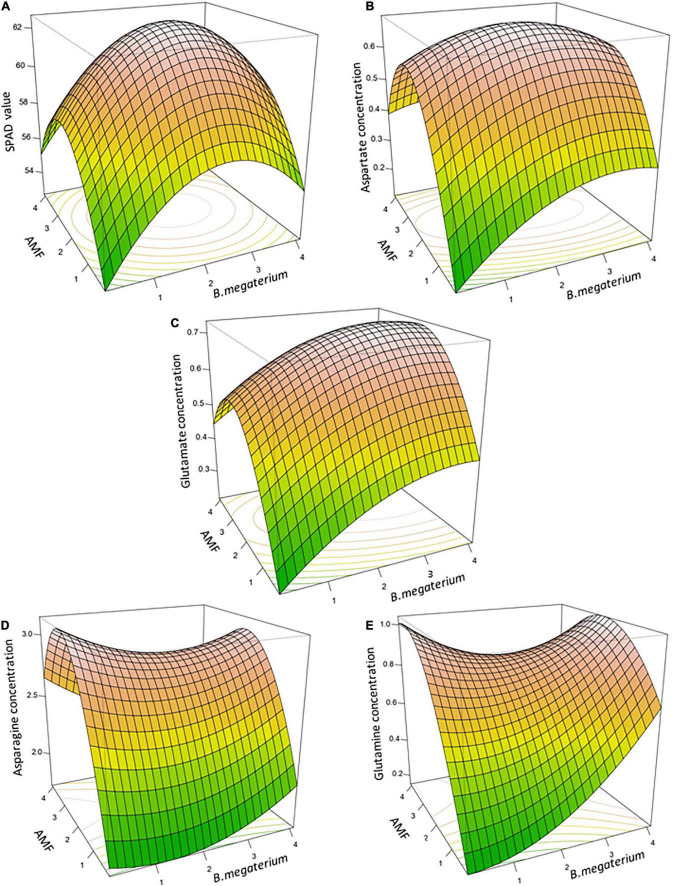
The three-dimensional response surface plots for interactive effects of *B. megaterium* and AMF co-inoculation on SPAD value **(A)**, aspartate concentration **(B)**, glutamate concentration **(C)**, asparagine concentration **(D)**, and glutamine concentration **(E)** in maize.

The final regression analysis equations were used for plotting 3D response surface and contour plots which represented the interaction of the input factors—AMF and *B. megaterium*—to trace the stationary point of each variable for the desired response (Figure 2 and Supplementary Figure 1). This stationary point was a maximum for SPAD value, aspartate, and glutamate, indicating a maximum response of these profiles obtained with mycorrhizal and bacterial-specific doses (Figure 2). The RSM optimized values for maximum SPAD value (62.66) were 2.27 ka ha^–1^ for AMF and 2.35 ka ha^–1^ for *B. megaterium*. Moreover, 2.22 and 2.24 ka ha^–1^ indicated the optimized doses of mycorrhizal and bacterial inocula to reach the highest aspartate content (0.69), while 2.41 ka ha^–1^ and 3.31 ka ha^–1^ obtained the maximum glutamate concentration. Concerning asparagine and glutamine, the stationary point was represented by a saddle point (2.91 and 0.85), which consisted of an inflection point of the response surface and was, respectively, achieved with 2.40 ka ha^–1^ and 2.36 ka ha^–1^, and with 2.99 ka ha^–1^ and 2.03 ka ha^–1^ of AMF and *B. megaterium* inocula. The contour plots of the fitted response surfaces allowed us to visualize the behavior of the fitted surface around the stationary point (Supplementary Figure 2). Each plot displayed a color image overlaid by the contour lines of constant responses in a two-dimensional space.

## Discussion

The choice of the best AMF-bacteria combination to improve NUE and thus ensure efficient and sustainable food production has become a burning question in the perspective of developing new strategies for the ecological transition of agriculture (Agnolucci et al., 2019). *B. megaterium* and AMF are both supposed to be potential bio-fertilizer agents able to play a key role in plant growth promotion. Their single and dual inoculation has revealed beneficial emerging properties in plant symbiosis, mostly translated into enhanced plant growth, yield, and abiotic stress tolerance (Ortiz et al., 2015; Khalid et al., 2017). RSM allows testing of multiple factors with complex interactions, modeling the system mathematically and using a limited number of experimental trials. However, despite the presence of studies based on the application of RSM models to improve plant growth, yield, and NUE, the statistical optimization of the best performing inoculum which can boost plant N acquisition is still limited in literature (Peng et al., 2014; Gundi et al., 2018; Naili et al., 2018; Mazumdar et al., 2021).

Several studies have shed light on the ability of AMF or PGPR to increase plant NUE, reporting higher grain and biomass values, as well as higher levels of NUtE, in different crops. In maize, clear evidence of improved NUE have been observed following inoculation with bacterial strains of *P. fluorescens* S3X and *C. necator* 1C2 (Pereira et al., 2020), *Bacillus megaterium* (Ganugi et al., 2022), and other. Concerning AMF, positive results were reported by single inoculation with *R. irregulare* BEG72 and *F. mosseae* BEG234, and coupled application of *Rhizophagus irregularis* and *Bacillus* spp. (Adesemoye et al., 2008).

Nevertheless, understanding the specific mycorrhizal and bacterial synergic action requires considerable study since PGPR–AMF affinity and colonization efficiency seem to be strongly related to the fungus species and origin. Marulanda-Aguirre et al. (2008) found different results for *B. megaterium* when inoculated on lettuce (*Lactuca sativa* L.) coupled with *Glomus constrictum* autochthonous, *G.constrictum* from a collection or commercial *G.intraradices*. In addition, this study highlights how an optimized mycorrhizal-to-bacterial inoculation rate on maize seeds at planting might be a success factor for the establishment and effectiveness of an AMF–PGPR symbiosis. Interestingly, our optimization aimed at enhancing maize NUE revealed similar RSM values for maximum SPAD, aspartate, and glutamate concentrations, which correspond to the intermediate doses of AMF and *B. megaterium*. Together with indicating a treatment effect on these NUE indices, this point confirmed the tripartite synergy between plants, mycorrhizae, and microbial communities for plant nutrition. Such synergy can be translated into a significant increase in N acquisition compared with the single inoculation of AMF or *B. megaterium* (Hestrin et al., 2019). However, the identification of saddle points for asparagine and glutamine pointed to the complexity of microorganisms’ interactions. The presence of such intricate microbial interactions indicates the need for further deep analyses, focused on experimental runs at points along this path, using the observed response values for guidance on where to locate the next factorial experiment (Lenth, 2009; Jan et al., 2021).

Despite the lack of significant treatment effect on most of the agronomic parameters, our data revealed interesting trends which is worth exploring in the future. On the one hand, it seems that maize plant biomass and biomass N-uptake could be increased by the concomitant action of AMF and *B. megaterium* at > 2.1. On the other hand, grain yield, grain N-uptake, and NHI showed a tendency to be increased preferentially by the single action of either AMF or *B. megaterium*, while lower values were registered under co-inoculation conditions. This latter statement was further corroborated by the fact that NHI (which was close to being significantly affected by the treatments) had the highest values with 4.1 kg ha^–1^ of AMF or *B. megaterium*, alternatively. These results suggest that (i) combining AMF and *B. megaterium* at a significant dose (> 2 kg ha^–1^) may have the potential to enhance plant tissues growth toward vegetative biomass, and (ii) if AMF and *B. megaterium* are inoculated alone, it is likely that grain-related agronomic parameters could benefit more than biomass-related ones. In fact, distinct effects provided by AMF, PGPR, and their co-inoculation in terms of drought resistance and essential oil yield have been previously reported in myrtle (Azizi et al., 2021). Similarly, it was reported that symbiotic efficiency may be hindered by interaction(s) with other biofertilizers together with being impacted by agro-practices (Kuila and Ghosh, 2022). In another work, Pacheco et al., evaluated the effect of AMF and *Pseudomonas putida* (PSB) single and co-inoculation on P uptake, productivity, and P concentration in maize (Pacheco et al., 2021). Interestingly, these authors reported that the microbial inoculants enhanced plant P uptake, that the presence of PSB increased biomass per unit of P taken up, and that the microbial inoculants altered P allocation within the plant, reducing grain P concentration (Pacheco et al., 2021). This distinct effect of the microbial inoculants on biomass production and nutrients allocation agrees with the results from Lozano Olivério Salvador and co-workers, who observed specific effects in terms of dry weight, symbiotic efficiency, chlorophyll content, and nitrogen accumulation when AMF or rhizobacteria were applied with compost to soybean (Salvador et al., 2022).

Despite the fact that mechanisms underlying the effect of single and combined inoculum are still unknown, amino acid metabolism represents an interesting target for crop NUE improvement, being actively involved in plant NUtE (Dellero, 2020). In particular, the glutamine synthetase/glutamate:2-oxoglutarate aminotransferase (GS/GOGAT) cycle represents the major route for plant ammonia assimilation, followed by asparagine synthetase (ASN) and glutamate dehydrogenase (GDH) (Harrison et al., 2000). Accordingly, experimental studies on maize have pointed out the increased concentration of aspartate and glutamate following *R. irregularis* and *Gigaspora margarita* inoculation (Matsumura et al., 2013; Hu and Chen, 2020), while the enhanced activity of ammonium assimilating enzymes (GS and GDH) have been detected with *Azospirillum* bacterial treatment (Ribaudo et al., 2001).

Here, for the first time, we reported how different rates of mycorrhiza-to-*Bacillus* co-inoculation may be considered—to some extent—as a driver of differential *N* partitioning between grain and vegetative biomass in the maize plant. This should be considered to address future studies to shed light on how such biotic interactions may steer plant physiology toward grain rather than vegetative tissues *N* accumulation.

Nevertheless, it should be stated some important aspects of the experiment. At first, it must be pointed out that the *Aegis Sym irriga*^®^ product is predominantly but not totally made by AMF, being associated—as the most commercial inocula—to other bacteria. For this reason, the effect of this inoculum on plant-based indices of NUE, as well as that obtained following its interaction with the PGPR product, is not necessarily ascribable to the exclusive presence of mycorrhiza. Second, to further complicate matters, plant responses should be studied under a wide range of soil-climate conditions, including different soil types and soil fertility, and temperate to dry climates. Particularly, physico-chemical soil properties, such as nutrient availability, pH, structure, organic matter content, and texture, can affect the release of plant root exudates and, consequently, the availability and the interactions with soil microbial communities (Neumann et al., 2014). It is likely that plant-microbe symbiosis is profoundly influenced by external environmental conditions, namely, temperature and moisture (Cheng et al., 2019). As consequence, it should be clarified that our results are strictly related to a specific pedoclimatic experimental context and, for this reason, maximum and saddle points for each NUE index cannot be certainly considered as absolute values exactly reproducible under different plant growth conditions.

Equally, the lack of significant effects in some agronomic indices may be reasonable because of the peculiar temperate and fertile conditions underlying the study, which is why it would be unreasonable to draw a conclusion about a lack of general effect of microbial inocula on the plant NUE. Notwithstanding, the application of prediction models may considerably contribute to advancements in the agricultural sector, particularly, under challenging factors interaction conditions.

## Data availability statement

The original contributions presented in this study are included in the article/Supplementary material, further inquiries can be directed to the corresponding author.

## Author contributions

PG, AF, VT, and LL conceived the ideas and designed the methodology. PG, AF, GR, and PB collected the data. PG, GR, and PB analyzed the data. PG, AF, PB, VT, and LL wrote the original draft. AF and LL critically revised the original draft. All the authors gave final approval for the publication.

## References

[B1] Anas M. Liao F. Verma K. K. Sarwar M. A. Mahmood A. Chen Z. L. (2020). Fate of nitrogen in agriculture and environment: agronomic, ecophysiological and molecular approaches to improve nitrogen use efficiency. *Biol. Res.* 53, 1–20. 10.1186/s40659-020-00312-4 33066819 PMC7565752

[B2] Adesemoye A. O. Torbert H. A. Kloepper J. W. (2008). Enhanced plant nutrient use efficiency with PGPR and AMF in an integrated nutrient management system. *Can. J. Microbiol.* 54 876–886. 10.1139/W08-081 18923557

[B3] Agnolucci M. Avio L. Pepe A. Turrini A. Cristani C. Bonini P. (2019). Bacteria associated with a commercial mycorrhizal inoculum: Community composition and multifunctional activity as assessed by illumina sequencing and culture-dependent tools. *Front. Plant Sci.* 9:1956. 10.3389/fpls.2018.01956 30693008 PMC6339933

[B4] Agnolucci M. Battini F. Cristani C. Giovannetti M. (2015). Diverse bacterial communities are recruited on spores of different arbuscular mycorrhizal fungal isolates. *Biol. Fertil. Soils* 51 379–389. 10.1007/s00374-014-0989-5

[B5] Ahmed M. Rauf M. Mukhtar Z. Saeed N. A. (2017). Excessive use of nitrogenous fertilizers: An unawareness causing serious threats to environment and human health. *Environ. Sci. Pollut. Res.* 24 26983–26987. 10.1007/s11356-017-0589-7 29139074

[B6] Azizi S. Tabari Kouchaksaraei M. Hadian J. Fallah Nosrat Abad A. R. Modarres Sanavi S. A. M. Ammer C. (2021). Dual inoculations of arbuscular mycorrhizal fungi and plant growth-promoting rhizobacteria boost drought resistance and essential oil yield of common myrtle. *For. Ecol. Manage.* 497:119478. 10.1016/j.foreco.2021.119478

[B7] Cheng Y. T. Zhang L. He S. Y. (2019). Plant-microbe interactions facing environmental challenge. *Cell Host Microbe* 26 183–192. 10.1016/j.chom.2019.07.009 31415751 PMC6697056

[B8] Congreves K. A. Otchere O. Ferland D. Farzadfar S. Williams S. Arcand M. M. (2021). Nitrogen use efficiency definitions of today and tomorrow. *Front. Plant Sci.* 12:637108. 10.3389/fpls.2021.637108 34177975 PMC8220819

[B9] Courty P. E. Smith P. Koegel S. Redecker D. Wipf D. (2015). Inorganic nitrogen uptake and transport in beneficial plant root-microbe interactions. *CRC. Crit. Rev. Plant Sci.* 34 4–16. 10.1080/07352689.2014.897897

[B10] Dalla Costa M. Rech T. D. Primieri S. Pigozzi B. G. Werner S. S. Stürmer S. L. (2021). Inoculation with isolates of arbuscular mycorrhizal fungi influences growth, nutrient use efficiency and gas exchange traits in micropropagated apple rootstock ‘Marubakaido.’ *Plant Cell. Tissue Organ Cult.* 145 89–99. 10.1007/s11240-020-01994-0

[B11] Dellero Y. (2020). Manipulating amino acid metabolism to improve crop nitrogen use efficiency for a sustainable agriculture. *Front. Plant Sci.* 11:602548. 10.3389/fpls.2020.602548 33329673 PMC7733991

[B12] Di Benedetto N. A. Corbo M. R. Campaniello D. Cataldi M. P. Bevilacqua A. Sinigaglia M. (2017). The role of plant growth promoting bacteria in improving nitrogen use efficiency for sustainable crop production: A focus on wheat. *AIMS Microbiol.* 3 413–434. 10.3934/microbiol.2017.3.413 31294169 PMC6604983

[B13] Galloway J. N. Winiwarter W. Leip A. Leach A. M. Bleeker A. Erisman J. W. (2014). Nitrogen footprints: Past, present and future. *Environ. Res. Lett.* 9:115003. 10.1088/1748-9326/9/11/115003

[B14] Ganugi P. Fiorini A. Ardenti F. Caffi T. Bonini P. Taskin E. (2022). Nitrogen use efficiency, rhizosphere bacterial community, and root metabolome reprogramming due to maize seed treatment with microbial biostimulants. *Physiol. Plant.* 174 1–15. 10.1111/ppl.13679 35362106 PMC9324912

[B15] Garcia K. Doidy J. Zimmermann S. D. Wipf D. Courty P. E. (2016). Take a trip through the plant and fungal transportome of mycorrhiza. *Trends Plant Sci.* 21 937–950. 10.1016/j.tplants.2016.07.010 27514454

[B16] Giovannini L. Palla M. Agnolucci M. Avio L. Sbrana C. Turrini A. (2020). Arbuscular mycorrhizal fungi and associated microbiota as plant biostimulants: Research strategies for the selection of the best performing inocula. *Agronomy* 10:106. 10.3390/agronomy10010108

[B17] Gundi J. S. Santos M. S. Oliveira A. L. M. Nogueira M. A. Hungria M. (2018). Development of liquid inoculants for strains of Rhizobium tropici group using response surface methodology. *Afr J Biotechnol* 17, 411–421. 10.5897/AJB2018.16389

[B18] Harrison J. Brugière N. Phillipson B. Ferrario-Mery S. Becker T. Limami A. (2000). Manipulating the pathway of ammonia assimilation through genetic 399 engineering and breeding: Consequences to plant physiology and plant development. *Plant Soil* 221 81–93. 10.1023/A:1004715720043

[B19] Hestrin R. Hammer E. C. Mueller C. W. Lehmann J. (2019). Synergies between mycorrhizal fungi and soil microbial communities increase plant nitrogen acquisition. *Commun. Biol.* 2:233. 10.1038/s42003-019-0481-8 31263777 PMC6588552

[B20] Hu Y. Chen B. (2020). Arbuscular mycorrhiza induced putrescine degradation into γ-aminobutyric acid, malic acid accumulation, and improvement of nitrogen assimilation in roots of water-stressed maize plants. *Mycorrhiza* 30 329–339. 10.1007/s00572-020-00952-0 32253571

[B21] Jan R. Asaf S. Numan M. Lubna Kim K. M. (2021). Plant secondary metabolite biosynthesis and transcriptional regulation in response to biotic and abiotic stress conditions. *Agronomy* 11:968. 10.3390/agronomy11050968

[B22] Khalid M. Hassani D. Bilal M. Asad F. Huang D. (2017). Influence of bio-fertilizer containing beneficial fungi and rhizospheric bacteria on health promoting compounds and antioxidant activity of *Spinacia oleracea* L. *Bot. Stud.* 58:35. 10.1186/s40529-017-0189-3 28815474 PMC5559411

[B23] Koegel S. Mieulet D. Baday S. Chatagnier O. Lehmann M. F. Wiemken A. (2017). Phylogenetic, structural, and functional characterization of AMT3; an ammonium transporter induced by mycorrhization among model grasses. *Mycorrhiza* 27 695–708. 10.1007/s00572-017-0786-8 28667402

[B24] Kollah B. Patra A. K. Mohanty S. R. (2016). Aquatic microphylla Azolla: A perspective paradigm for sustainable agriculture, environment and global climate change. *Environ. Sci. Pollut. Res.* 23 4358–4369. 10.1007/s11356-015-5857-9 26697861

[B25] Kuila D. Ghosh S. (2022). Aspects, problems and utilization of Arbuscular Mycorrhizal (AM) application as bio-fertilizer in sustainable agriculture. *Curr. Res. Microb. Sci.* 3:100107. 10.1016/j.crmicr.2022.100107 35169758 PMC8829076

[B26] Kumar V. Kumar P. Khan A. (2020). Optimization of PGPR and silicon fertilization using response surface methodology for enhanced growth, yield and biochemical parameters of French bean (*Phaseolus vulgaris* L.) under saline stress. *Biocatal. Agric. Biotechnol.* 23:101463. 10.1016/j.bcab.2019.101463

[B27] Lenth R. V. (2009). Response-surface methods in R, using RSM. *J. Stat. Softw.* 32 1–17. 10.18637/jss.v032.i07

[B28] López-Bellido R. J. López-Bellido L. (2001). Efficiency of nitrogen in wheat under Mediterranean conditions: Effect of tillage, crop rotation and N fertilization. *F. Crop. Res.* 71 31–46. 10.1016/S0378-4290(01)00146-0

[B29] Maris S. C. Abalos D. Capra F. Moscatelli G. Scaglia F. Reyes G. E. C. (2021). Strong potential of slurry application timing and method to reduce N losses in a permanent grassland. *Agricult. Ecosyst. Environ.* 311:107329. 10.1016/j.agee.2021.107329

[B30] Marulanda-Aguirre A. Azcón R. Ruiz-Lozano J. M. Aroca R. (2008). Differential effects of a *Bacillus megaterium* strain on *Lactuca sativa* plant growth depending on the origin of the arbuscular mycorrhizal fungus coinoculated: Physiologic and biochemical traits. *J. Plant Growth Regul.* 27 10–18. 10.1007/s00344-007-9024-5

[B31] Matsumura A. Taniguchi S. Yamawaki K. Hattori R. Tarui A. Yano K. (2013). Nitrogen uptake from amino acids in maize through arbuscular mycorrhizal symbiosis. *Am. J. Plant Sci.* 4 2290–2294. 10.4236/ajps.2013.412283

[B32] Mazumdar D. Saha S. Ghoshet S. (2021). RSM based optimization of plant growth promoting rhizobacteria and nitrogen dosage for enhanced growth and yield of mustard (*Brassica campestris* L.). *J. Plant Nutr.* 44 2228–2244. 10.1080/01904167.2021.1889585

[B33] Moreau D. Bardgett R. D. Finlay R. D. Jones D. L. Philippot L. (2019). A plant perspective on nitrogen cycling in the rhizosphere. *Funct. Ecol.* 33 540–552. 10.1111/1365-2435.13303

[B34] Mus F. Crook M. B. Garcia K. Garcia Costas A. Geddes B. A. Kouri E. D. (2016). Symbiotic nitrogen fixation and the challenges to its extension to nonlegumes. *Appl. Environ. Microbiol.* 82, 3698–3710. 10.1128/AEM.01055-16 27084023 PMC4907175

[B35] Naili F. Neifar M. Elhidri D. Cherif H. Bejaoui B. Aroua M. (2018). Optimization of the effect of PGPR-based biofertlizer on wheat growth and yield. *Biometric. Biostat. Int. J.* 7 226–232. 10.15406/bbij.2018.07.00213

[B36] Neumann G. Bott S. Ohler M. A. Mock H. P. Lippmann R. Grosch R. (2014). Root exudation and root development of lettuce (*Lactuca sativa* l. Cv. Tizian) as affected by different soils. *Front. Microbiol.* 5:2. 10.3389/fmicb.2014.00002 24478764 PMC3901204

[B37] Oldroyd G. E. D. Murray J. D. Poole P. S. Downie J. A. (2011). The rules of engagement in the legume-rhizobial symbiosis. *Annu. Rev. Genet.* 45 119–144. 10.1146/annurev-genet-110410-132549 21838550

[B38] Ortiz N. Armada E. Duque E. Roldán A. Azcón R. (2015). Contribution of arbuscular mycorrhizal fungi and/or bacteria to enhancing plant drought tolerance under natural soil conditions: Effectiveness of autochthonous or allochthonous strains. *J. Plant Physiol.* 174 87–96. 10.1016/j.jplph.2014.08.019 25462971

[B39] Pacheco I. Ferreira R. Correia P. Carvalho L. Dias T. Cruza C. (2021). Microbial consortium increases maize productivity and reduces grain phosphorus concentration under field conditions. *Saudi J. Biol. Sci.* 28 232–237. 10.1016/j.sjbs.2020.09.053 33424302 PMC7785415

[B40] Paul K. Saha C. Nag M. Mandal D. Naiya H. Sen D. (2020). A tripartite interaction among the basidiomycete rhodotorula mucilaginosa, N2-fixing endobacteria, and rice improves plant nitrogen nutrition. *Plant Cell* 32 486–507. 10.1105/tpc.19.00385 31757927 PMC7008492

[B41] Peng S. Buresh R. J. Huang J. Yang J. Zou Y. Zhong X. (2006). Strategies for overcoming low agronomic nitrogen use efficiency in irrigated rice systems in China. *F. Crop. Res.* 96 37–47. 10.1016/j.fcr.2005.05.004

[B42] Peng Y. He Y. Wu Z. Lu J. Li C. S. (2014). Screening and optimization of low-cost medium for *Pseudomonas putida* Rs-198 culture using RSM. *Braz. J. Microbiol.* 45 1229–1237. 10.1590/s1517-83822014000400013 25763026 PMC4323295

[B43] Pereira S. I. A. Abreu D. Moreira H. Vega A. Castro P. M. L. (2020). Plant growth-promoting rhizobacteria (PGPR) improve the growth and nutrient use efficiency in maize (*Zea mays* L.) under water deficit conditions. *Heliyon* 6:e05106. 10.1016/j.heliyon.2020.e05106 33083600 PMC7550905

[B44] Qu Q. Zhang Z. Peijnenburg W. J. G. M. Liu W. Lu T. Hu B. (2020). Rhizosphere microbiome assembly and its impact on plant growth. *J. Agric. Food Chem.* 68, 5024–5038. 32255613 10.1021/acs.jafc.0c00073

[B45] R Core Team (2013). *R: A Language and Environment for Statistical Computing*.

[B46] Ribaudo C. Rondanini D. P. Curá J. A. Fraschina A. A. (2001). Response of *Zea mays* to the inoculation with *Azospirillum* on nitrogen metabolism under greenhouse conditions. *Biol. Plant.* 44 631–634. 10.1023/A:1013779712106

[B47] Rosenblueth M. Ormeño-Orrillo E. López-López A. Rogel M. A. Reyes-Hernández B. J. Martínez-Romero J. C. (2018). Nitrogen fixation in cereals. *Front. Microbiol.* 9:1794. 10.3389/fmicb.2018.01794 30140262 PMC6095057

[B48] Salvador G. L. O. Araújo F. F. de Araujo Pereira A. P. Bonifácio A. Ferreira Araujo A. S. (2022). Rhizobacteria and arbuscular mycorrhizal fungus presented distinct and specific effects on soybean growth when inoculated with organic compost. *Rhizosphere* 22:100513. 10.1016/j.rhisph.2022.100513

[B49] Santi C. Bogusz D. Franche C. (2013). Biological nitrogen fixation in non-legume plants. *Ann. Bot.* 111 743–767. 10.1093/aob/mct048 23478942 PMC3631332

[B50] Tajini F. Trabelsi M. Drevon J. J. (2012). Combined inoculation with *Glomus intraradices* and *Rhizobium tropici* CIAT899 increases phosphorus use efficiency for symbiotic nitrogen fixation in common bean (*Phaseolus vulgaris* L.). *Saudi J. Biol. Sci.* 19 157–163. 10.1016/j.sjbs.2011.11.003 23961175 PMC3730892

[B51] The S. V. Snyder R. Tegeder M. (2021). Targeting nitrogen metabolism and transport processes to improve plant nitrogen use efficiency. *Front. Plant Sci.* 11:628366. 10.3389/fpls.2020.628366 33732269 PMC7957077

[B52] Udvardi M. Poole P. S. (2013). Transport and metabolism in legume-rhizobia symbioses. *Annu. Rev. Plant Biol.* 64 781–805. 10.1146/annurev-arplant-050312-120235 23451778

[B53] van Bueren L. E. T. Struik P. C. (2017). Diverse concepts of breeding for nitrogen use efficiency. A review. *Agron. Sustain. Dev.* 37:50. 10.1007/s13593-017-0457-3

[B54] van Dam N. M. Bouwmeester H. J. (2016). Metabolomics in the rhizosphere: Tapping into belowground chemical communication. *Trends Plant Sci.* 21 256–265. 10.1016/j.tplants.2016.01.008 26832948

[B55] Verzeaux J. Hirel B. Dubois F. Lea P. J. Tétu T. (2017). Agricultural practices to improve nitrogen use efficiency through the use of arbuscular mycorrhizae: Basic and agronomic aspects. *Plant Sci.* 264 48–56. 10.1016/j.plantsci.2017.08.004 28969802

[B56] Wickham H. Chang W. (2007). *ggplot2: An implementation of the grammar of graphics. GNU general public license.* Available online at: https://cran.r-project.org/web/packages/ggplot2 (accessed May 03, 2022).

